# Early preventive administration of inhaled tobramycin in severe polytrauma

**DOI:** 10.1186/cc14201

**Published:** 2015-03-16

**Authors:** A Kuzovlev, A Shabanov, T Chernenkaya, V Moroz, A Goloubev

**Affiliations:** 1 V.A. Negovsky Research Institute of General Reanimatology, Moscow, Russia; 2 N.V. Sklifosofsky Research Institute, Moscow, Russia

## Introduction

Nosocomial pneumonia (NP) occurs in 30 to 50% of multiple trauma patients. It is mostly caused by multiresistant Gram-negative bacteria. Use of inhaled antibiotics as adjuncts to systemic antibiotics presents a great outlook for the prevention of NP in multiple trauma patients. The aim of the study was to evaluate the efficacy of early administration of inhaled tobtamycin (IT) as an adjunct to systemic antibiotics for the prevention of NP in polytrauma.

## Methods

Fifty-four ICU mechanically ventilated patients with multiple trauma (ISS >30; car accident 55.6%; fall 29.6%; train accident 11.1%; domestic 3.7%) were enrolled in the single-center randomized trial. Groups were comparable in ISS, age, sex, type of trauma, and blood loss. Patients were randomized into two groups: Group 1 (*n *= 27), addition of IT to systemic antibiotics (ciprofloxacin 800 mg/day; metronidazol 1,500 mg/day); Group 2 (*n *= 27), only systemic antibiotics (same regimen). Inhaled tobramycin (300 mg twice daily via nebulizer) and systemic antibiotics were administered within the first 24 hours after ICU admission. After obtaining the results of bronchoalveolar lavage microbiology, the antibiotic regimen was switched according to the sensitivity. The primary outcome measure was new onset of NP and duration of ICU stay. Microbiological, X-ray, CPIS, signs of sepsis and oxygenation index were used as objective indicators of the clinical progress. The secondary outcome measure was 30-day mortality. Diagnosis of NP was made according to the standard clinical and CPIS criteria. The data were statistically analyzed by SPSS 11.5 (M, σ, Newman-Keuls test; chi-square-test *P *< 0.05).

## Results

Preventive administration of IT as an adjunct to systemic antibiotics was associated with a lower incidence of NP in group 1 (group 1 33.3%, group 2 66.7%, χ^2 ^= 6,000; *P *= 0.014) and a shorter duration of ICU stay (group 1 8.0 ± 4.6 days vs. 17.1 ± 18.4 days, *P *= 0.03). The mortality did not differ between groups: 11.1% in group 1 and 22.2% in group 2 (*P *≥0.99). On day 3 *Acinetobacter *spp. (30.5%), *K. pneumoniae *(22.0%), *B. cepacia *(13.2%) and *P. aeruginosa *(34.3%) were detected in BAL, there were no differences between groups. In group 1 CPIS remained stable and APACHE II decreased. CPIS and APACHE II were lower in group 1 on day 5 (*P *= 0.0004).

## Conclusion

Early administration of IT as an adjunct to systemic antibiotics is effective in prevention of NP in multiple trauma patients: it promotes decrease of NP incidence and decrease of ICU stay.

